# Recurrent Renal Cell Carcinoma with Synchronous Tumor Growth in Azygoesophageal Recess and Duodenum: A Rare Cause of Anemia and Upper Gastrointestinal Bleeding

**DOI:** 10.1155/2015/143934

**Published:** 2015-11-12

**Authors:** Vamshidhar R. Vootla, Muhammad Kashif, Masooma Niazi, Suresh K. Nayudu

**Affiliations:** ^1^Division of Gastroenterology, Department of Medicine, Bronx-Lebanon Hospital Center, 1650 Selwyn Avenue, Suite No. 10 C, Bronx, NY 10457, USA; ^2^Division of Pulmonary and Critical Care Medicine, Department of Medicine, Bronx-Lebanon Hospital Center, 1650 Selwyn Avenue, Suite No. 12 F, Bronx, NY 10457, USA; ^3^Department of Pathology, Bronx-Lebanon Hospital Center, 1650 Selwyn Avenue, Suite No. 10 C, Bronx, NY 10457, USA

## Abstract

Renal cell carcinoma (RCC) has potential to present with distant metastasis several years after complete resection. The common sites of metastases include the lungs, bones, liver, renal fossa, and brain. RCCs metastasize rarely to the duodenum, and duodenal metastasis presenting with acute gastrointestinal bleed is infrequently reported in literature. We present a case of synchronous presentation of duodenal and azygoesophageal metastasis manifesting as acute upper gastrointestinal bleeding, four years after undergoing nephrectomy for RCC. The patient underwent further workup and was treated with radiation. The synchronous presentation is rare and stresses the importance of searching for recurrence of RCC in patients presenting with acute gastrointestinal bleeding.

## 1. Introduction

Malignancy accounts for about 5% of upper gastrointestinal hemorrhage and may need surgical intervention in the majority of cases. Various segments of the upper gastrointestinal tract can be involved, but duodenal involvement is very rare.

RCC accounts for approximately 3% of all malignancies in adults leading to approximately 13,000 deaths annually in the United States of America. Renal cell carcinoma has a strong tendency to metastasize many years following surgical resection. Metastatic sites for RCC are the lungs, bone, liver, adrenal glands, and brain; however, gastrointestinal tract can be involved for solitary late recurrence in rare instances. Duodenal metastasis of RCC has rarely been reported in the literature, but synchronous tumor presence in azygoesophageal recess and duodenum presenting as melena was never reported in literature to the best of our knowledge.

## 2. Case Presentation

**Figure 1:**
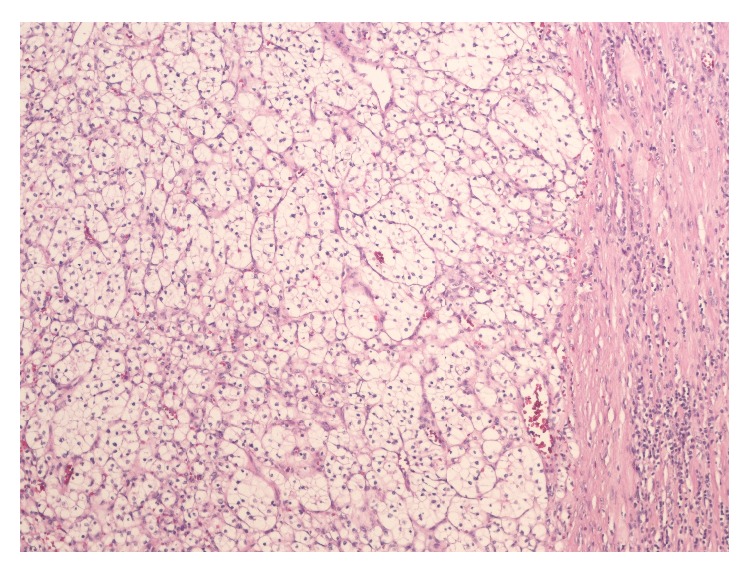
Kidney histopathology: renal cell carcinoma. Clear cell type, comprised of prominent delicate vasculature surrounding alveolar clusters of carcinoma cells.

We present the case of a 74-year-old man who presented to our primary care clinic with an episode of dark colored stool. He denied any other gastrointestinal symptoms at the time of presentation. He reported subjective weight loss in the last few months. He was diagnosed with RCC four years ago when he was noted to have an incidental renal mass on computerized tomography (CT) of the abdomen. A total nephrectomy was performed revealing renal carcinoma with clear cell subtype on histopathology (Figure 1). Fuhrman nuclear grade was I–III. The mass was confined to the renal capsule and on further workup staged as T2aN0cM0. Subsequently, he was followed up in the oncology clinic during which imaging studies did not reveal any residual or recurrent tumor.

He was also known to have hypertension, type II diabetes mellitus, coronary artery disease, asthma, gout, chronic kidney disease stage II, and benign ethnic leukopenia. He was a former smoker with a 20-pack-year history of tobacco smoking. His father was diagnosed with colon cancer at the age of 74.

On initial evaluation his vitals were temperature 98.6 F, pulse 88/minute, respiratory rate 15/minute, and blood pressure 130/88 mm Hg. He was obese with BMI of 30 and had conjunctival pallor on general physical examination. There was bilateral air entry on auscultation of lungs with no adventitious sounds. Precordial examination revealed normal heart sounds with no murmur, rub, or gallop. His abdomen was soft without any tenderness with no palpable masses. Rectal examination was significant for decreased sphincter tone and external hemorrhoids. Laboratory evaluation showed iron deficiency anemia, with ferritin level of 11 micrograms/liter. Serum chemistry showed elevated creatinine of 1.9 gm/dL consistent with his chronic kidney disease. His liver chemistries were within normal limits.

**Figure 2:**
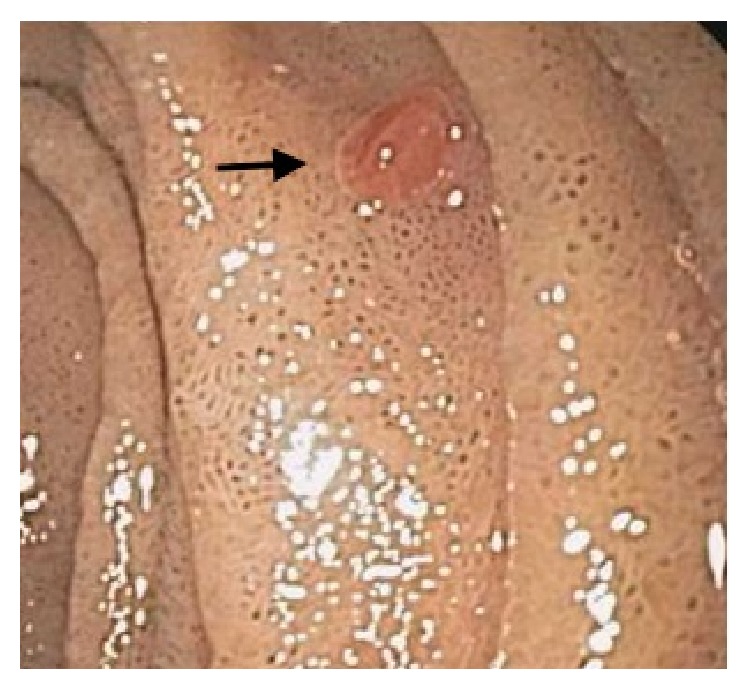
Upper gastrointestinal endoscopy revealing a nodule (arrow) in the second part of the duodenum.

**Figure 3:**
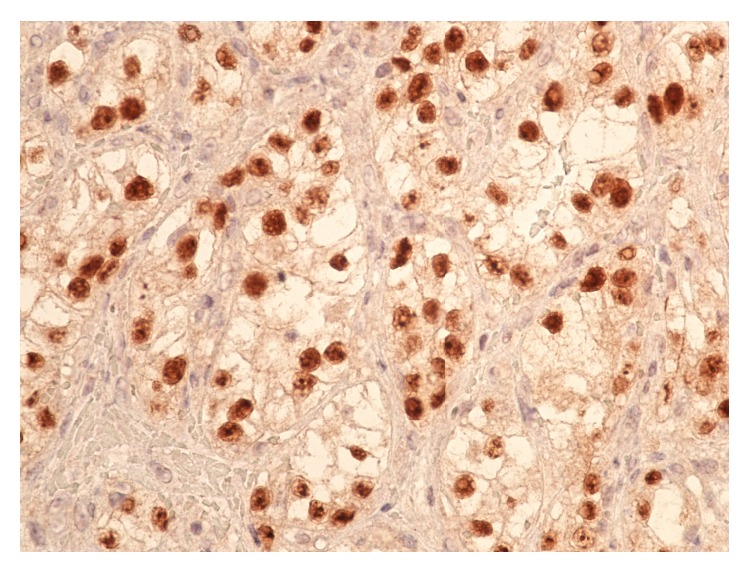
Duodenal mucosa with metastatic alveolar clusters of clear cell renal carcinoma. Tumor cells strongly immunoreactive to PAX8 intranuclear immunostain.

He underwent emergent upper gastrointestinal endoscopy and colonoscopy. Colonoscopy showed colonic diverticulosis and a few hyperplastic polyps in the rectum and sigmoid colon. Endoscopy showed duodenal nodule that was biopsied and histopathology revealed duodenal mucosa showing nests and clusters of neoplastic cells with clear cytoplasm in the lamina propria, consistent with metaplastic RCC (Figures 2 and 3). Additional upper endoscopic findings were peptic duodenitis, chronic gastritis, and esophageal candidiasis.

**Figure 4:**
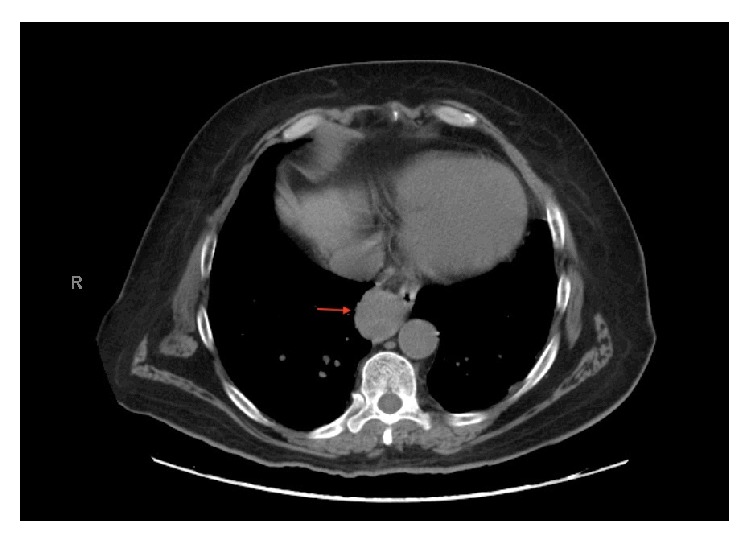
CT scan showing a 3.5 cm azygoesophageal recess mass closely approximating the esophagus.

Patient underwent workup for staging with CT scan of the abdomen and pelvis showing normal appearing right kidney. A 3.5-centimeter mass was noted in the azygoesophageal recess closely approximating the esophagus (Figure 4). There was no evidence of brain metastasis on Magnetic Resonance Imaging (MRI) of the brain. Positron Emission Tomography (PET) scan was done for further evaluation of the mass, which showed a 3.5 cm mass in the azygoesophageal recess with abnormal hyper metabolic activity confirming malignant origin. An endoscopic ultrasound (EUS) was performed with fine needle aspiration (FNA) cytology of the mass, which revealed RCC. Retrospective review of the images performed for initial staging showed no nodules in the recess.

Multidisciplinary team including surgery, oncology, and radiation oncology evaluated the case to plan further management. In view of metastatic disease and recurrent unstable angina episodes suggestive of severe coronary artery disease, patient was deemed not a suitable candidate for surgical resection. He was started on radiation therapy and subsequently being followed up in the medical oncology clinic.

## 3. Discussion

RCC is known to be associated with late recurrence of metastatic disease. Levy et al. reported that 23.8% of patients with RCC developed metastasis years after radical nephrectomy in T2 N0 M0 patients [1]. This risk of development of recurrent malignant disease was high during the first three postoperative years. RCC has potential to metastasize to almost any site but the most common sites are the lung (75%), lymph nodes (36%), bone (20%), liver (18%), adrenal glands, kidney, brain, heart, spleen, GI tract, and skin. Approximately 4% of RCCs metastasize to the GI tract and account for 7.1% of all metastatic tumors to the small intestine [2].

Duodenal metastasis is an uncommon presentation of RCC [3]. There have been 28 cases of isolated metastasis to the duodenum reported so far [4]. The majority of patients are found to have duodenal metastasis within the first three years after nephrectomy, though it can be seen even after several years [5]. The routes of spread can be (i) peritoneal dissemination, (ii) direct spread from an intra-abdominal malignancy, (iii) hematogenous spread, and (iv) lymphatic spread. In the duodenum, periampullary region is the most common site of metastasis followed by the duodenal bulb. Though solitary duodenal metastasis has been reported, duodenal metastasis in the setting of widespread nodal and visceral involvement has been found to be more common [6]. Duodenal metastasis has been reported to present with varied clinical presentations including abdominal pain, anemia, gastrointestinal bleeding, duodenal obstruction, or intussusception [5, 7, 8]. On endoscopy these lesions can be seen as polypoid mass, nodules, or submucosal mass with ulceration [9]. If lesions are in the submucosal position, regular biopsy specimens may not be sufficient to clinch the diagnosis and may need aggressive biopsy techniques using jumbo forceps or surgical biopsy to obtain sufficient tissue for diagnosis [10].

Our case is unique with synchronous presentation of a duodenal and azygoesophageal metastasis presenting as melena. Management of metastatic RCC is dependent on the site. The treatment options for solitary duodenal RCC metastasis depend upon the extent and location of the lesion and range from metastasectomy to Whipple procedure. In cases of overt GI bleeding attributed to duodenal metastasis, interventional embolization has been reported to control the bleeding effectively. Isolated solitary metastasis of RCC has better prognosis than widespread metastasis, which is highly resistant to chemotherapy and radiotherapy, and the treatment of metastatic RCC currently remains ineffective. Sunitinib is currently the most widely used oral vascular endothelial growth factor receptor kinase inhibitor for the initial treatment of metastatic clear cell RCC [11]. Although there have been advances in chemotherapy, the median survival time of patients with metastatic RCC is 6–12months, and the 5-year survival rate is only 9% [12].

## 4. Conclusion

If patients with history of RCC have any gastrointestinal symptoms including occult or overt bleeding, obstructive symptoms, or iron deficiency anemia, the possibility of metastasis to duodenal or gastrointestinal tract involvement should be kept in mind nevertheless with a history of nephrectomy. Such patients should be investigated at the earliest with appropriate radiological studies, upper endoscopy, and colonoscopy. Awareness of this entity and a high index of suspicion on the part of the treating physician and pathologist would help in proper diagnosis and treatment.

## Abbreviations

RCC: Renal cell carcinoma
GI: Gastrointestinal
CT: Computerized tomography
MRI: Magnetic Resonance Imaging
EUS: Endoscopic ultrasound.
